# Responsible and innovative AI for mental health care: five priority themes

**DOI:** 10.1038/s44277-026-00068-x

**Published:** 2026-08-20

**Authors:** Martin P. Paulus, John Torous, Roy H. Perlis, Karthik V. Sarma, Olusola Ajilore, Sahib S. Khalsa, Carolyn I. Rodriguez

**Affiliations:** 1 Laureate Institute for Brain Research, Tulsa, OK USA; 2 Department of Psychiatry, Beth Israel Deaconess Medical Center, Harvard Medical School, Boston, MA USA; 3 Department of Psychiatry and Center for Quantitative Health, Massachusetts General Hospital; Harvard Medical School, Boston, MA USA; 4 Department of Psychiatry and Behavioral Sciences and Institute for Health Policy Studies, University of California, San Francisco, San Francisco, CA USA; 5 Department of Psychiatry, University of Illinois Chicago; College of Medicine, Chicago, IL USA; 6 Department of Psychiatry and Biobehavioral Sciences, Semel Institute for Neuroscience and Human Behavior, David Geffen School of Medicine, University of California, Los Angeles, CA USA; 7 Department of Psychiatry and Behavioral Sciences, Stanford University School of Medicine, Stanford, CA USA

**Keywords:** Health services, Scientific community

## Abstract

Artificial intelligence (AI) has entered psychiatry at scale, yet its clinical impact remains constrained by a sizable gap between technical validation and real-world implementation. The central barriers are no longer computational, but infrastructural: unreliable measurement systems, incomplete governance frameworks, and insufficient standards for clinical evidence and integration. This paper synthesizes insights from a 2026 American College of Neuropsychopharmacology (ACNP) study group examining how to responsibly translate AI into clinical mental health care. Building on this perspective, we outline five priorities required for clinical impact. First, robust measurement and phenotyping infrastructure, such as reliable psychometrics, digital phenotyping, and standardized data pipelines, is essential for clinically meaningful AI. Second, the most immediate and scalable impact of AI lies in clinician-facing augmentation tools that reduce workflow burden, such as ambient documentation systems and structured decision-support pipelines, with important research to conduct here. Third, patient-facing AI interventions show promise but require rigorous safety evaluation, particularly for implicit suicide risk and heterogeneous treatment effects. Fourth, governance and equity frameworks must extend beyond privacy to address bias, digital literacy, and research integrity. Fifth, future progress requires moving beyond predictive models toward causal, mechanistic approaches to precision psychiatry that better inform treatment decisions and clinical action. Together, these priorities define a translational agenda for 2026 and beyond: AI in mental health will succeed not through model performance alone, but through disciplined integration into clinical workflows, measurement systems, and governance structures that ensure safety, equity, and real-world effectiveness.

## Lay Summary

This paper synthesizes insights from a 2026 American College of Neuropsychopharmacology (ACNP) study group examining how to responsibly translate AI into clinical mental health care. Building on this perspective, we outline five priorities required for clinical impact. These priorities define a translational agenda for 2026 and beyond: AI in mental health will succeed not through model performance alone, but through disciplined integration into clinical workflows, measurement systems, and governance structures that ensure safety, equity, and real-world effectiveness.

## Introduction — Why this paper, why now?

Artificial intelligence (AI) tools—from deep learning neuroimaging classifiers to large language model (LLM) powered chatbots—are moving rapidly from proof of concept to pilot deployment in psychiatry; yet most still sit in the “validation and implementation gap.” To address that gap, this paper emerges from a study group convened at the 2026 American College of Neuropsychopharmacology (ACNP) annual conference, which brought together mental health experts whose careers span therapeutic discovery and real-world implementation. Study group participants represented diverse research methods, fields, and approaches, as well as multiple career stages, among them clinician-scientist, computational neuroscientist, ethicist-regulator, early-career data scientist, patient-advocate-researcher, and industry collaborator. The group examined how psychiatry as a field can accelerate responsible, equitable translation of AI into routine mental health care while avoiding the pitfalls of bias, opacity, and workflow strain. AI may contribute to gains in diagnostic precision, continuous care, and operational efficiency, but premature adoption risks harm and erosion of public trust.

**Fig. 1:**
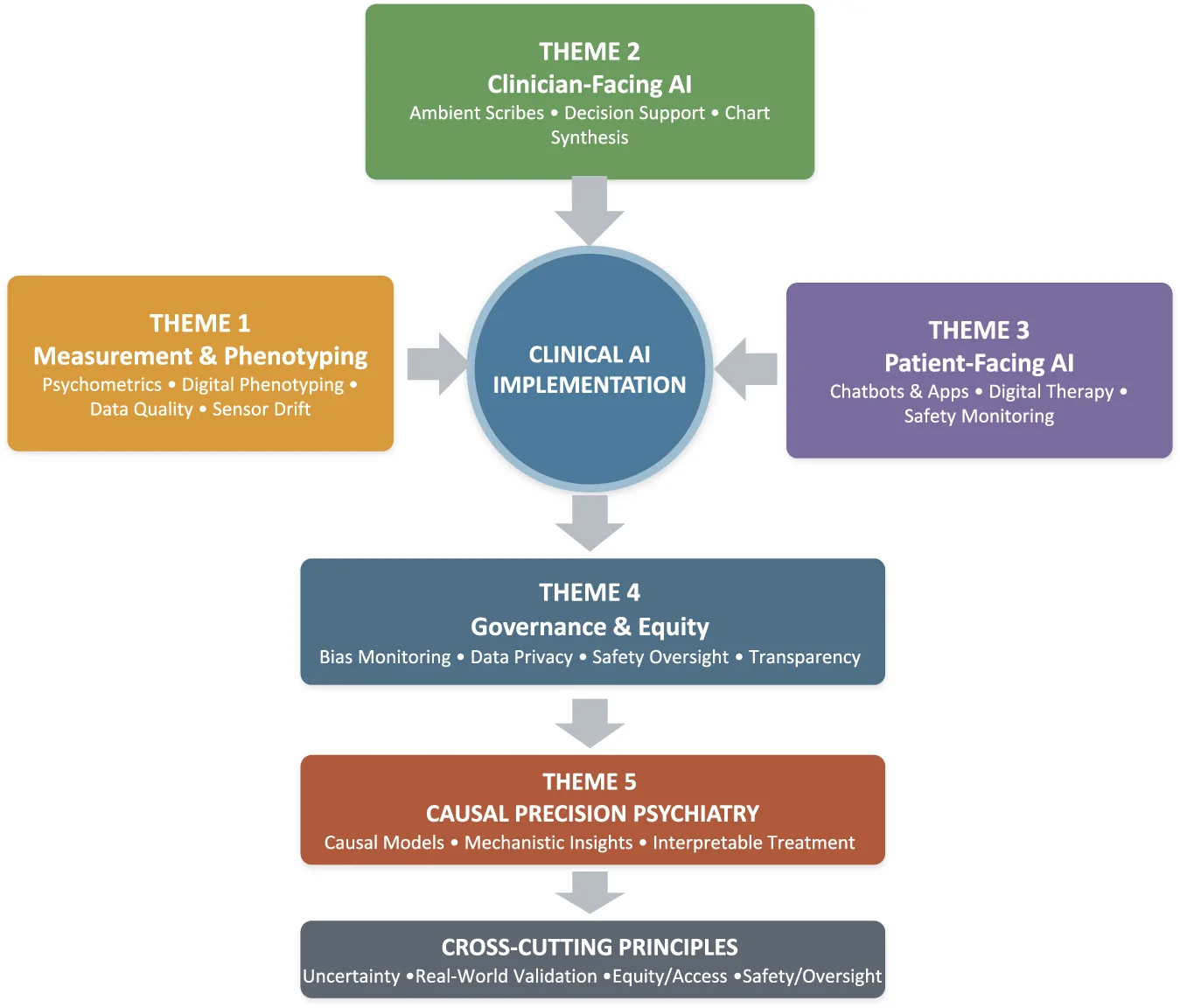
Five Priority Themes for Responsible and Innovative AI for Mental Health Care.

This study group identified and presented themes for actionable strategies to design, evaluate, and deploy AI tools that improve patient outcomes and advance the science of mental health (see Fig. 1 and Table 1). Specifically, the group concluded that this transition requires (i) measurement systems with explicit ground-truth strategies and uncertainty handling, (ii) workflow-native augmentation with auditability and oversight, (iii) patient-facing tools evaluated as clinical interventions with both short-term and longitudinal safety endpoints, (iv) governance that protects patients, communities, and the scientific commons from predictable AI-driven distortions, and (v) mechanistic and causal precision psychiatry. Here, ground truth refers to the reference standard used to judge model correctness; uncertainty handling refers to methods for communicating low confidence, missing evidence, or ambiguity, and workflow-native augmentation refers to AI support embedded inside the clinician’s existing task sequence rather than added as a separate tool.

**Table 1:** Five themes for actionable strategies to design, evaluate, and deploy AI tools that improve patient outcomes and advance the science of mental health.

*Theme*	*Primary Focus*	*Key Challenges*	*Actionable Recommendations*
Theme 1: Measurement & Phenotyping	The primary constraint for mental health AI in 2026 is measurement infrastructure, rather than model capacity.	Unstable and loosely defined phenotypes yield brittle predictions. Imputing missing-not-at-random data can induce optimism bias and degrade utility.	Develop infrastructure with validated phenotypes, ontology mappings, and drift monitoring. Treat severity scoring as important data but alone not the gold standard.
Theme 2: Clinician-Facing Augmentation	The dominant near-term clinical use of AI is documentation via LLM-powered ambient scribes.	Generative tools risk introducing hallucinated facts, false certainty, and narrative homogenization.	Engineer tools as workflow-integrated clinical decision support systems. Specify implementation roles, training, monitoring, and pause/rollback authority. Implement structured red-teaming to probe psychiatric edge cases.
Theme 3: Patient-Facing Interventions	Direct behavioral health AI applications are highly scalable but carry the most risk of failure.	Human-like conversational competence does not equate to clinical efficacy. Models frequently fail to identify implicit safety cues like hopelessness.	Frame AI chatbots as adjunctive tools, not equivalents to psychotherapy. Test active ingredients, mediators, longitudinal safety, and implicit-risk detection. Apply a tiered certification model where evidentiary burdens scale with risk.
Theme 4: Governance & Equity	Governance must actively manage patient safety, equity, and research ecosystem health.	Latent constructs and high-stakes outcomes make governance stricter. Lower digital literacy can degrade performance. High-quality synthetic data introduces risks of scientific fraud. Compute and energy demands may create environmental-equity burdens.	Mandate real-world and behavioral testing beyond standard engineering benchmarks. Implement public performance dashboards, dataset-demographic reporting, incident-response procedures, and use-case-level justification for right-sized models.
Theme 5: Mechanistic Psychiatry	Moving precision psychiatry toward actionable, interpretable, and causally robust models.	Prediction remains necessary but insufficient. Black-box machine learning often captures correlations rather than assumptions needed for treatment selection and counterfactual reasoning.	Develop generative, biophysically constrained models that provide testable hypotheses. Evaluate AI as a co-intervention by modeling interaction effects.

## Theme 1 — Measurement and phenotyping as infrastructure

The binding constraint for mental health AI in 2026 is less about model capacity and more about measurement infrastructure: what clinical target is being measured, how reliably it can be measured, and whether the measure is comparable across devices, sites, clinicians, and time [1]. A phenotype is the observable clinical, behavioral, biological, or functional target that an AI system is asked to measure, classify, or predict. In psychiatry, phenotypes may include symptom severity, functional impairment, suicide risk, treatment response, activity/sleep patterns, or clinician-rated mental status. The central measurement problem is that many of these targets are partly subjective, context dependent, and recorded differently across clinicians, devices, and health systems. Thus, the first question for mental health AI is not “which model performs best?” but “what target is the model learning, how reliable is that target, and does the target map onto a decision clinicians can act on?”

The first problem is psychometric reliability. Much of computational psychiatry still underspecifies core measurement properties, including test-retest reliability and parameter recovery, yielding a “stability gap” in which candidate biomarkers fluctuate despite minimal true clinical change [2]. A parameter that cannot be measured reproducibly cannot credibly support trial stratification, longitudinal monitoring, or treatment selection. At the same time, reliability must be interpreted in context: a measure designed to detect clinically meaningful state change can appear unreliable if practice effects, fatigue, time of day, or clinical context are not modeled [3]. The practical standard should therefore be explicit: measurement models must separate trait from state variance, examine context and practice effects, and treat reliability, calibration, and recovery as primary endpoints rather than optional appendices.

The second problem is digital phenotyping, meaning the use of passively or actively collected digital signals, such as sleep/activity, mobility, device interaction, speech, or physiology, to characterize clinically relevant behavior. These streams can anchor phenotypes beyond narrative text, but only if their limitations are treated as design constraints rather than after-the-fact caveats [4, 5]. Battery drain, fragmented device ecosystems, iOS/Android constraints, and algorithmic drift can all change the meaning of the same feature over time. Standardized acquisition protocols, device-aware calibration, and drift monitoring should be treated as statistical process control for mental health measurement [4]. Likewise, missing-not-at-random patterns are part of the phenotype-data generating process: patients with greater symptoms or older hardware may be precisely the patients whose data collapse. Imputation that ignores this pattern can induce optimism bias and reduce clinical utility [4, 6].

The third problem is semantic harmonization: psychiatry remains a “Tower of Babel” in which clinical phenomenology, biology, and computational constructs are only loosely aligned. Dimensional frameworks are useful, but they do not become engineering specifications until translated into machine-readable constructs with explicit bridging statements: what is being measured, how it is extracted, and what evidence counts as support. There are scientific process and transparent methods that harmonize cross-platform feature definitions, for example, so that “a step,” “a home stay,” or “sleep regularity” means the same thing across time, despite platform [7, 8]. In short, the near-term deliverable is not simply a new model class; it is a measurement infrastructure package with validated phenotypes, ontology mappings, multisite calibration, drift monitoring, missingness audits, and outputs that remain clinically interpretable and decision-relevant.

The ACNP study group operationalized the discussion by focusing on factors that determine deployability: measurement-based care, severity scoring, and the construction of ground truth [9, 10]. In clinical AI, ground truth should not be assumed to exist as a single obvious label; it must often be built through adjudication, multi-source evidence, and explicit uncertainty rules. For example, measurement-based care is a realistic near-term application but cannot rely on a single scale or label as the ground truth, given that such does not exist today in clinical psychiatry. Rather, the system must identify the specific evidence supporting each symptom score, abstain when evidence is insufficient, and trigger predefined escalation pathways, such as clinician review or structured follow-up questions.

The group also emphasized the *incremental validity bar* for multimodal severity estimation: combining narrative text with measurable data (sleep/activity, speech markers, adherence proxies) is attractive, but it only helps if (i) it improves calibration and downstream clinical utility beyond text alone, and (ii) missing-not-at-random patterns are explicitly characterized and stress-tested, particularly in more symptomatic patients. This is an important inferential point: multimodal fusion can amplify bias when one modality is systematically missing in the very populations where measurement matters most, and the “best” fused model on complete cases can be the worst model in real deployment. A related thread concerned quantifying the mental status exam. The appropriate 2026 strategy is “decision separation:” quantitative markers (speech latency, coherence, affective variability, thought-form indicators) may inform hypotheses, monitoring, and training feedback, but should not replace clinician judgment absent prospective validation tied to clear clinical actions—and tied to an infrastructure that monitors drift, missingness, and rubric faithfulness as first-class endpoints.

## Theme 2 — Clinician-facing augmentation and workflow-native clinical decision support

In 2026, the dominant clinical use of AI in psychiatry is not diagnosis or treatment selection, but documentation, especially LLM-powered ambient scribes that reduce friction in clinical workflows. This reality underscores a central translational lesson: clinician-facing AI currently delivers measurable benefit only when it is workflow-integrated, not merely workflow-adjacent. A tool layered on top of clinical work may add another dashboard, inbox, or alert; a workflow-integrated tool changes the task sequence itself by delivering the right information at the point where a clinician can act, documenting who is accountable for the action, and creating a record that can be audited.

Clinician-facing AI represents the most credible near-term pathway to measurable benefit in mental health, but only when implemented as a sociotechnical system rather than a model deployment. The translational claim is deliberately narrow: LLM-enabled tools can reduce friction (documentation, chart synthesis, guideline retrieval) and improve decision quality (risk stratification, treatment planning support) only when outputs are tied to explicit clinical actions, bounded by scope, and embedded within governance that enforces uncertainty communication, auditing, and lifecycle monitoring. The appropriate evaluation metric is therefore not the fluency of generated text, but the clinical workflow and decisions it changes.

An established implementation scaffold for the next step, clinical decision support (CDS), is the “Five Rights:” right information, right person, right format, right channel, and right time [11, 12]. Generative models require two technical updates to this framework. First, “right information” becomes probabilistic: confidence and uncertainty displays are integral rather than optional. Second, “right format” often shifts from interruptive alerts toward inline drafts optimized for clinician review. In practice, psychiatric LLM-CDS should be specified as a constrained pipeline (retrieval → evidence-grounded synthesis → uncertainty labeling → action linkage) rather than deployed as a general conversational agent. A near-term operational priority is the development of psychiatry-specific LLM-CDS specifications defining permissible tasks, required uncertainty displays, explicit clinical action linkages (e.g., to support patient screening, safety planning, medication reconciliation), with a longer-term goal of developing and testing escalation pathways for high-risk contexts.

Ambient clinical intelligence (ACI)—systems that use ambient audio capture and natural language processing to generate structured clinical documentation—illustrates the clearest current example of workflow-native augmentation. ACI addresses a well-established bottleneck: documentation burden, which may absorb clinician time, attention, and promote burnout. In the case of current workflows, the latest cross-specialty evidence suggests a reduction of documentation-associated burden over time savings [13]. Clinically consequential failure modes that could complicate include hallucinated or misattributed facts, inappropriate diagnostic certainty, and narrative homogenization that suppresses nuance essential for longitudinal assessment and risk evaluation. These errors can propagate through copy-forward documentation practices, influencing coding, downstream clinical decisions, and future model training. However, in psychiatry, mental status discernment and case formulation are more complex tasks requiring interpretive judgments rather than simple transcriptions. Of the available studies, the limitations predominate, including difficulty with complex cases [14], potential underestimation of suicide risks [15, 16], and evaluation of clinical vignettes as opposed to real-world cases [17].

The primary design objective is to calibrate trust—aligning user reliance directly with system capability—rather than merely maximizing explainability. CDS must support calibrated skepticism by helping clinicians determine when to rely on—or override—system outputs. Structured red-teaming is therefore methodologically essential [18]. Independent adversarial testing should explicitly probe psychiatric edge cases, including implicit suicidality, psychosis content, medication misuse, coercion risk, and adolescent vulnerability. Post-deployment monitoring should track drift and emergent failure patterns rather than relying on static validation.

A related engineering tradeoff concerns prompting strategy. Prompting methods that maximize accuracy may reduce explanation quality, whereas simpler prompting approaches often produce more interpretable rationales with only modest accuracy tradeoffs. In clinical settings, the optimal configuration is likely not defined by accuracy alone but by dual performance standards: decision-quality metrics (accuracy, calibration, clinically meaningful error taxonomy) and audit-quality metrics (evidence grounding, rationale interpretability, and clinician verifiability).

Implementation risk is also influenced by a persistent platform compliance gap. Health systems may outpace their own governance capacity when procuring AI applications, which leaves monitoring, bias auditing, and incident response underdeveloped [19]. While comprehensive governance is ideal, resource constraints make fully centralized oversight difficult in practice. Emerging guidance emphasizes the need for shared governance models, including institutional oversight bodies, vendor accountability requirements, and standardized monitoring frameworks to distribute responsibility while maintaining safety [20, 21].

Feasibility is therefore a workforce and governance problem as much as a technical one. Practical deployment requires protected clinical leadership, implementation science expertise, informatics and AI/machine learning support, privacy/security and legal review, vendor management capacity, frontline clinical champions, and patient/community representation. Training must be role-specific: clinicians need digital literacy, ability to interpret uncertainty, override practices, and escalation procedures. Operational leaders need procurement, monitoring, and incident-response standards. Because these functions are expensive and scarce, especially in under-resourced systems, scalable implementation will likely require shared governance templates, regional validation resources, and reusable monitoring infrastructure.

Value added must be defined in workflow terms and tested against the specific task sequence into which the AI system is integrated. For ACI, chart summarization, and guideline retrieval, success should be measured using pre-specified endpoints such as time saved, reduced omissions, improved documentation quality, improved downstream clinical decisions, and demonstrated reductions in clinician burnout, rather than satisfaction ratings alone. Oversight structures must also be explicit, with defined accountability for clinical safety, technical performance, equity monitoring, and operational management, including authority to pause or modify systems following safety regressions. Human factors considerations further require design constraints that mitigate automation bias, including uncertainty displays, counter-evidence presentation, and monitoring of acceptance and override patterns. To operationalize these requirements, health systems must execute a phased deployment: initiate integration within clinics utilizing highly protocolized care (e.g., Transcranial Magnetic Stimulation) and systematically scale the architecture conditional on validated performance.

Taken together, clinician-facing LLM deployment in mental health should be approached as a phase-gated clinical engineering program rather than an innovation sprint. Safe and effective implementation requires: (i) workflow-native use cases with explicit action linkage, (ii) evidence-grounded outputs with uncertainty and abstention, (iii) red-teamed safety evaluation, (iv) governance infrastructure proportionate to system risk, and (v) protections to preserve clinical record integrity. The challenge is less so in the ideals but more in the execution, as the need for agreement and cooperation both within and outside the field is substantial, and history has proven challenging. When these conditions are met, clinician-facing augmentation offers the most scalable and lowest-risk pathway to near-term impact. When they are not, the apparent efficiency gains risk degrading decision quality and documentation integrity in ways that are difficult to detect but consequential over time.

## Theme 3 — Patient-facing AI interventions: from engagement demos to clinical therapeutics

Patient-facing AI-driven applications that directly provide behavioral health interventions were seen by the ACNP study group to be simultaneously the most scalable and the most potentially failure-prone innovation for care delivery in 2026. The central concern was the potential for conflation of conversational competence and clinical efficacy. That is, models that have very human-like interactions may not necessarily have the desired clinical impact.

Empirically, current general-purpose LLMs are not convincingly safe as stand-alone tools for self-diagnosis or treatment guidance, but they do show promise (through assessment of knowledge and information processing) for use as a building block. In one evaluation with prompts in the style of the United States Medical Licensing Examination (USMLE), GPT-4 was judged correct in only 31% (29/94), and agreement between rater groups was 34% [22]. However, in a different study using boards-style psychiatric questions, the same model answered 84% correctly, and yet when asked to self-assess its confidence, no correlation was found between self-assessed confidence and actual correctness [23]. In an assessment of psychiatric diagnostic reasoning, the model exhibited a sensitivity of 76%, but a positive predictive value (PPV) of only 39%, representing substantial overdiagnosis [17]. Early simulation studies and case reports suggest that generalist models are at risk of providing inappropriate mental health advice [24, 25]. This evidence does not imply LLMs are unusable; it implies that unconstrained “ask-me-anything” mental health applications are structurally misaligned with safety and correctness.

More focused clinical evidence is emerging but still nascent. A recent randomized clinical trial of a hybrid chatbot (Therabot [26]) using prior-generation but specialized language models reported symptom reductions. However, gaps in durability, generalizability, cohort diversity, identification of active therapeutic components, and, particularly, concerns that the waitlist control may not allow for the clear establishment of clinically significant impact will likely limit its impact. The implication for 2026 is that the high degree of conversational competence of these models does not directly imply accuracy or efficacy, and indeed, “chatbot efficacy” should not be treated as a single claim. A major concern is the continued investigation of such interventions against wait-list control, rather than active interventions.

The study group noted that current AI chatbot agents are often framed as “psychotherapy” or as wellness interventions that could substitute for psychotherapy. This framing is too broad. The more precise questions are whether AI systems can reproduce or support specific active ingredients of psychotherapy, such as therapeutic alliance, empathic responsiveness, collaborative goal setting, and skills practice, whether they can influence validated mediators of change, such as negative schemas, avoidance, emotion regulation, or behavioral activation, and whether these ingredients can be deployed in a manner that is convincingly safe and beneficent. Opel and Breakspear [27] emphasize that chatbots can mimic empathic language but do not experience empathy or emotion; the American Psychological Association similarly notes that AI agents may not be able to establish a genuine, two-way therapeutic alliance [28]. AI chatbots should therefore not be framed as equivalents to psychotherapy. They are better conceptualized as tools whose active ingredients, mediators, safety profile, and clinical indications require direct testing.

Safety is the decisive constraint for patient-facing systems because they operate outside clinician supervision, at scale, and in emotionally charged contexts. The most actionable translation of this concern is to specify safety in testable behaviors. Importantly, safety failures in mental health are often implicit rather than explicit. For example, models may respond appropriately to explicit suicidal ideation prompts but fail to recognize implicit cues, such as hopelessness, perceived burdensomeness, escalating isolation, or coded references. This creates a safety false-negative risk in which a human clinician might escalate concern but the model does not. One safety-relevant existing effort is DeepSuiMind, a developing dataset intended to improve implicit suicidal ideation recognition [16]. Numerous additional simulation efforts [29, 30] are attempting to provide testing frameworks; however, few peer-reviewed efforts have been published [31], and it is essential to evaluate their coverage of realistic patient phenotypes and very long conversations, including 100-turn or 1000-turn interactions. Patient-facing tools must be evaluated on their performance in detecting and intervening upon implicit risk, not merely on their response to clear, explicit crisis inputs.

Safety evaluation must also extend beyond acute test prompts. Patient-facing chatbots should undergo longitudinal monitoring for potential harms or safety guardrail degradations that may emerge over repeated interactions, including delayed recognition of suicidality, reinforcement of hopelessness or maladaptive beliefs, excessive reliance on the agent, reduced help-seeking, particularly during very long conversations [32]. In this sense, patient-facing AI requires “digital pharmacovigilance:” systematic monitoring of adverse events, near misses, escalation failures, and subgroup-specific harms after deployment.

The study group also noted that a second methodological requirement is to abandon the expectation that a single patient-facing AI intervention will benefit “on average” across the highly heterogeneous risk strata seen in behavioral health. For example, in one suicide prevention digital therapeutic trial [33], the pre-planned whole-study analysis did not find a significant effect, but a high-risk subgroup (prior attempts) was reported to show a 58.3% reduction in suicide attempt rate. Such interpretation should be disciplined: subgroup findings require caution, but they also indicate that effect heterogeneity is plausible. In 2026, the correct stance is to treat patient-facing AI as personalized and targeted (matched to risk phenotype and context) rather than universally deployable.

Effect sizes in lower-risk populations are likely to be modest, and that must be accepted as the baseline expectation. One synthesis of AI chatbot interventions reported meta-analytic evidence in adolescents showing small-to-moderate benefits for depression (SMD − 0.43) and anxiety (SMD − 0.37), while also noting engagement fragility and drop-off absent personalization [34]. This combination suggests a pragmatic agenda: optimize retention and adherence mechanisms, but also prioritize endpoints beyond symptom scales (function, self-efficacy, service engagement).

The study group noted that given the rapid adoption of generative technologies in mental health (such as ambient scribes, EHR summarization tools, psychotherapy assistant tools, and potential autonomous chatbot agents), a new concern about “what is real” (vs generated hallucinations) and the downstream effects of this concern on records and science has arisen. In the best case, this creates noise; in the worst case, it drives false reassurance, inappropriate de-escalation, or misleading self-concepts. The correct 2026 response is not to ban generation, but to require provenance where possible (i.e., linking content to primary data) and to implement “digital pharmacovigilance:” systematic monitoring for adverse events.

The empirical constraints of AI-based interventions map directly onto the study group’s perspective that a structured framework is required. One proposed example is a tiered certification model: Tier 0 informational psychoeducation; Tier 1 guided self-management; Tier 2 adjunctive therapeutic support in diagnosed cohorts; and Tier 3 tools that can influence clinical actions (e.g., triage, safety planning prompts). The evidentiary burden should scale with risk: from usability and content fidelity (Tier 0) to randomized controlled trials (RCT)/pragmatic evidence with safety endpoints and escalation validation (Tiers 2–3). The “do no harm” principle should be made measurable: (i) no fabricated clinical facts, (ii) calibrated uncertainty and abstention, (iii) conservative escalation on risk cues (including implicit cues), (iv) privacy protection, and (v) validated refusal behavior for unsafe or out-of-scope requests.

## Theme 4 — Governance, equity, and research-ecosystem health

In mental health AI, governance is the methodological scaffold that determines whether systems improve care or amplify harm. In 2026, governance must operate across three coupled layers: (i) patient safety and accountability, (ii) equity, and (iii) research ecosystem incentives that shape what science is pursued and what evidence is produced.

The baseline governance approach in mental health must be stricter than in many other clinical domains because targets are often latent constructs, error costs are high, and outcomes can perpetuate stigma and structural bias [20, 21, 35]. By latent constructs, we mean clinically meaningful but indirectly observed targets (e.g., depression severity or impulsivity, or psychosis risk) that are inferred from symptoms, behavior, clinician judgment, or records rather than measured as directly as a laboratory value. Governance is stricter in this setting because disagreement can arise not only from model error but also from ambiguity in the construct itself. While some guardrails can be treated as engineering requirements—proactive risk identification, transparency/explainability, privacy/security, human oversight with explicit escalation pathways, continuous monitoring and updating, regulatory/ethical compliance, and clear accountability for failure—others cannot. Importantly, many guardrails are not static compliance artifacts; they are themselves behavioral variables that can materially change how a model interacts with distressed users and clinicians. Mental health systems therefore need more than basic engineering testing or benchmarking; they need behavioral testing, real-world testing, and monitoring for safety regressions after model updates. The importance of this real-world testing was highlighted in a 2026 study, which reported that AI systems with impressive performance for diagnosis and prognosis in engineering tests actually degraded to no better than chance performance when used with actual people who used the AI tools in ways not intended (e.g. made errors, did not provide correct prompts) [36].

Equity is also critical to realizing AI’s potential in mental healthcare. Digital literacy remains a primary barrier to digital health adoption, and both patients and clinical teams need practical training and support [37–39]. The association between workforce shortage and hospital AI non-adoption may reflect limited implementation capacity rather than limited need [40]. An implementation-capacity paradox may exist: systems with the greatest mental health workforce gaps may have the least protected clinical, informatics, and governance capacity to procure, validate, monitor, and sustain AI safely [40]. This observation supports targeted digital-literacy and implementation training for psychiatrists, therapists, nurses, and operational leaders. It also argues against assuming that AI improves care simply because it is deployed; prior hospital experience in sepsis and emergency-department workflows illustrates that AI can fail to improve outcomes when workflow integration, trust calibration, and accountability are insufficient. Equity concerns may also extend to data ownership and governance as AI systems increasingly request and reuse patient health data. How these data are used, reused, and represented remains largely unknown, raising issues of both equity and trust in mental health AI.

Regulatory efforts appear mixed in their support for safer and more transparent uses of AI. The Office of the National Coordinator for Health Information Technology (ONC) Health Data, Technology, and Interoperability (HTI-1) rule requires transparency for predictive decision-support interventions (including disclosure of training data, bias testing, and demographic source attributes), which is scheduled to sunset in 2026 [41]. The January 2026 FDA guidance on low-risk medical devices and clinical recommendations indicates that the agency will consider many uses of AI, particularly for mental health, outside the scope of formal regulation [42, 43]. Current heterogeneity in state mental health AI laws only adds to the challenge. Without support from classical regulatory channels, new grassroots efforts must be developed to operationalize basic requirements (model cards, dataset documentation, bias evaluations), monitoring plans (drift and subgroup performance), and incident response procedures (rollback authority and accountability for regressions). A 2026 governance response should establish concrete accountability mechanisms, including mandatory reporting on dataset composition and demographic coverage, preregistered replication targets, public performance dashboards stratified by subgroup, and funding incentives tied to inclusion of high-need, low-data populations.

The ACNP study group also extended governance concerns to sustainability, record integrity, and the risks of scientific fraud introduced by high-quality synthetic text and data. Sustainability is also an equity issue. Large-scale AI systems can impose compute, water, and energy demands, and the infrastructure built to support data centers may concentrate environmental and health burdens in communities that are already under-resourced. Mental health AI governance should therefore require use-case-level justification for model size, reporting of compute/energy costs where feasible, and preference for right-sized or local models when comparable clinical performance can be achieved with lower resource intensity. Additional boundary conditions for trustworthy deployment include provenance markers and traceable citations for clinical assertions to protect longitudinal record integrity, dataset provenance checks, and explicit labeling of synthetic data to reduce fraud risk. The overarching implication is that “keeping the model moral” is not a model property; it is an institutional accountability regime with defined allowable behaviors, enforced refusal/escalation policies, and clear responsibility when updates regress safety or integrity.

## Theme Five — mechanistic and causal precision psychiatry

A central barrier in precision psychiatry is not only the generation of predictors. Better predictors, endpoints, and validation cohorts remain necessary; however, prediction alone is insufficient when the clinical question is which intervention should be selected, for whom, under what assumptions, and with what expected benefit or harm. Decision-grade models must support a specific clinical action, remain valid under changes in care, and communicate their evidentiary basis and uncertainty. Pure risk prediction can aid triage, but it is often policy-dependent and can fail once interventions change the data-generating process. Clinicians ultimately need causal and mechanistic models that estimate which intervention improves outcomes for which patients, and through which modifiable processes. The field is saturated with black-box machine learning, which often captures correlations. Clinically credible precision psychiatry requires models that distinguish prediction from explanation and make their assumptions testable.

The ACNP study group agreed that associations should be seen as starting points, not mechanistic claims. Effective workflows must include replication, falsification tests, sensitivity analyses, and interventional validation. Without clear estimates and confounding controls, predictors may mislead clinicians into treating correlations as modifiable targets. Accurate prediction can lack clinical impact, while causal models enable counterfactual reasoning essential for treatment selection.

Next-generation causal AI should pair general-purpose foundation models with topic-specific causal models for high-stakes decisions. Causal claims around treatment selection around often context-specific (eg unique presentation of the patient at that time and their preferences for treatment) and so making universal causal claims is challenging and unreliable without narrow validation. Recommendations should be evidence-anchored and should abstain or escalate when evidence is sparse, rather than producing confident but incorrent guidance. The group stressed that AI should be evaluated as a co-intervention, acknowledging its potential to influence patient engagement and clinician decisions. Trials must model interaction effects (e.g., AI × treatment modality) as primary analyses. A recent preliminary study using this design by Benrimoh et al. demonstrated that an AI-enhanced clinical decision support tool for antidepressant treatment selection had higher remission rates and a faster time to remission than an active control group [44]. Therefore, future trials evaluating AI-delivered therapies must employ active comparators, explicitly benchmarking novel systems against both domain-specific clinical agents and general-purpose foundation models.

Neuroimaging studies present a common use case that often blurs mechanism and correlation. Explainability, measured by stability, method agreement, robustness, and clinical linkage, is necessary for trust and regulation. Productivity gains in neuroimaging should be judged by reproducibility and error reduction, emphasizing workflow documentation and provenance tracking over unsourced causal interpretations. There are several examples emerging in the literature where causal AI is incorporated into model design to enhance the explainability and robustness of the mode. These include models in which domain knowledge about specific regions of interest (ROIs) and connectivity patterns are built-in to enhance interpretability [45, 46]. This theme concluded that “AI progress” in mental health means building mechanistic and causal pipelines that distinguish prediction from explanation and specify assumptions for intervention.

## Conclusion

Considered in aggregate, the five priority themes examined in this paper all reflect the insight that the primary barriers to responsible AI deployment in mental health are not computational but infrastructural. Model architectures have outpaced the measurement systems, governance frameworks, and evidence-collection methods needed to make their outputs clinically meaningful. We note in Theme 1 that absent validated and harmonized phenotypes, even high-performing models tend to learn less performant proxies rather than constructs. Themes 2 and 3 demonstrate that this fragility manifests differently depending on the deployment context. Clinician-facing tools degrade documentation integrity and downstream decision-making via hallucination as well as homogenization of descriptions. Patient-facing tools may lack therapeutic efficacy and risk missing important safety signals. Theme 4 explicitly states the need for governance structures to enforce equity and research integrity. The very populations most in need of improved mental health care bear the greatest risk of harm, such as those with lower digital literacy and those served by under-resourced systems. Theme 5 emphasizes the limitations of risk scores—even accurate ones, which may lead clinicians to focus on metrics that do not truly lead to clinical benefit—and the need to move toward precision and causal psychiatry.

Across these themes, several cross-cutting principles also emerge. A specific concern is the handling of uncertainty. Rather than suppressing uncertainty, it must be clearly expressed regardless of the modality being tested. In addition, the context from which benchmarks emerge, and their limitations, must be emphasized. Results in a simulation are not necessarily reflective of real-world settings; for example; even real-world measures may change over time. A particular concern is the focus on average treatment effects, which can obscure the heterogeneity of outcomes across a population. The way in which AI tools will be applied must be considered a priori, rather than post hoc, particularly in thinking about appropriate testing and governance.

These principles should serve to guide implementation, not simply to slow it. There are numerous opportunities beyond incorporating better measurement and documentation, as well as prospects for novel therapeutic tools. All of these require consideration of context and particular effort to ensure safety, not just efficacy. There is substantial risk that, without thoughtful processes in place, AI could exacerbate underlying disparities, amplifying differences in digital literacy and access to care. On the other hand, with careful adoption, AI can meaningfully augment the capacity of a mental health system under extraordinary strain.
